# Common Peroneal Nerve Compression Neuropathy Due to a Large Synovial Cyst From the Proximal Tibiofibular Joint in a Teenager

**DOI:** 10.7759/cureus.46562

**Published:** 2023-10-06

**Authors:** Mohamed A Khalefa, Shakir Hussain, Edwards C Bache

**Affiliations:** 1 Trauma and Orthopedics, The Royal Orthopedic Hospital, Birmingham, GBR; 2 Trauma and Orthopedics, Cairo University, Cairo, EGY; 3 Trauma and Orthopedics, Birmingham Children's Hospital, Birmingham, GBR

**Keywords:** rare tumors, ortho surgery, foot drop, knee ganglion cyst, common peroneal neuropathy

## Abstract

Ganglion cysts are very rare in the lower limb and when present, ganglion cysts rarely cause compression neuropathy at any site. Peripheral nerve sheath tumors as a whole, are also very rare and mostly presented as a painful lump along the nerve path.

Ganglion cysts are non-neoplastic gelatinous cysts, which lack true synovial lining. They can be divided into intraneural cysts which can be found within the epineurium of a peripheral nerve and lead to signs and symptoms of peripheral neuropathy or extraneural cysts which can develop from surrounding joints or tendon sheaths causing gradual nerve compression.

Intraneural tumors of common peroneal nerve (CPN) are widely reported in the literature with varying degrees of symptoms; however, there are only a few case reports describing CPN palsy due to extraneural cysts. We are reporting a rare case of atraumatic CPN palsy, which resulted in irreversible foot drop in a teenage boy who presented with right leg radiating calf and foot pain. We recommend prompt investigation and excision of the cyst to decompress the nerve to increase the chances of early recovery and favorable outcomes.

## Introduction

Ganglion cysts are a common presentation in orthopedic practice and mostly present as painless lumps. However, if they happen in close proximity to a nerve, they can present with pain and occasionally radiating nerve symptoms which are mostly mild and inconsistent. Some ganglions, however, can produce profound nerve symptoms and even nerve palsy, if they are large enough to cause nerve compression. The later presentation is rare and dependent on the site of the ganglion.

The most common ganglion compression neuropathy is the Ulnar nerve when the ganglion compresses the nerve in the cubital tunnel [1]. It is less commonly seen in the lower limb but there have been few case reports reporting common peroneal nerve (CPN) palsy in relation to ganglion cysts. The first case of compression neuropathy of CPN was reported by Sultan et al. in 1921 [2]. Ozden et al. [3] reported that extra-neural ganglion cysts can affect the Sciatic nerve, CPN, Sural Nerve, and Posterior Tibial Nerve. Intraneural ganglions of the CPN are very rare [4].

The most common site for CPN compression neuropathy is as it winds around the fibular neck which can result in atraumatic foot drop [5]. This situation poses difficulty in diagnosis and the majority of these patients can present quite late in the disease process i.e., with established complete foot drop. The recovery of the CPN depends on the extent of nerve injury at the time of decompression and the age of the patient [6].

We are reporting a rare case of CPN palsy due to compression by a ganglion cyst originating from the proximal tibiofibular joint. We emphasize prompt investigation and early decompression of the CPN to prevent permanent damage to the nerve.

## Case presentation

A 16-year-old boy was referred to a pediatric orthopedic surgeon with a foot drop presentation that developed over almost two years. The initial presentation was vague with mild pain around the knee radiating down the front of the lower leg. No history of recent trauma or swelling around the knee. Due to the slow progression of his symptoms, it was difficult for both the patient and his family to appreciate the gradual nature of his foot drop till it became very profound causing him to walk with a slapping foot gait.

The patient had an unremarkable past medical history. He had sustained an ipsilateral closed ankle fracture whilst playing football two years prior to his current presentation which was managed conservatively in plaster for six weeks.

He presented with a classic foot drop gait but with no significant wasting of the anterior compartment or calf muscles. He denied any knee or ankle joint tenderness. His knee, ankle, and subtalar joints were supple with a normal range of motion. Normal distal pulsations were present and there was no sensory loss. However, grading his motor function, he suffered from foot drop with complete loss of power in the anterior and lateral compartment muscles of the leg, with no dorsiflexion or eversion, but preserved inverters and plantar flexors. Detailed Medical Research Council (MRC) grading is outlined in Table 1.

**Table 1:** MRC grades at the time of presentation MRC - Medical Research Council

Muscle	MRC grade
Tibialis Anterior (Ankle Dorsiflexion)	0
Extensor Hallucis Longus (Big toe)	0/1
Extensor Digitorum Longus (toes)	1/2
Peroneal Longus and Brevis (evertors)	1/2
Tibialis Posterior (Ankle Plantarflexion)	5
Achille’s tendon (Plantar flexors)	5

Differential diagnosis

An initial differential diagnosis included causes for mononeuropathy. The possibility of plaster-related complications that may have led to CPN compression palsy such as an edge of a tight plaster cast or a missed compartment syndrome was considered. In addition, possible lumbar spine pathology affecting L4,5 nerve roots was also included in the working diagnosis.

Investigations

**Figure 1:**
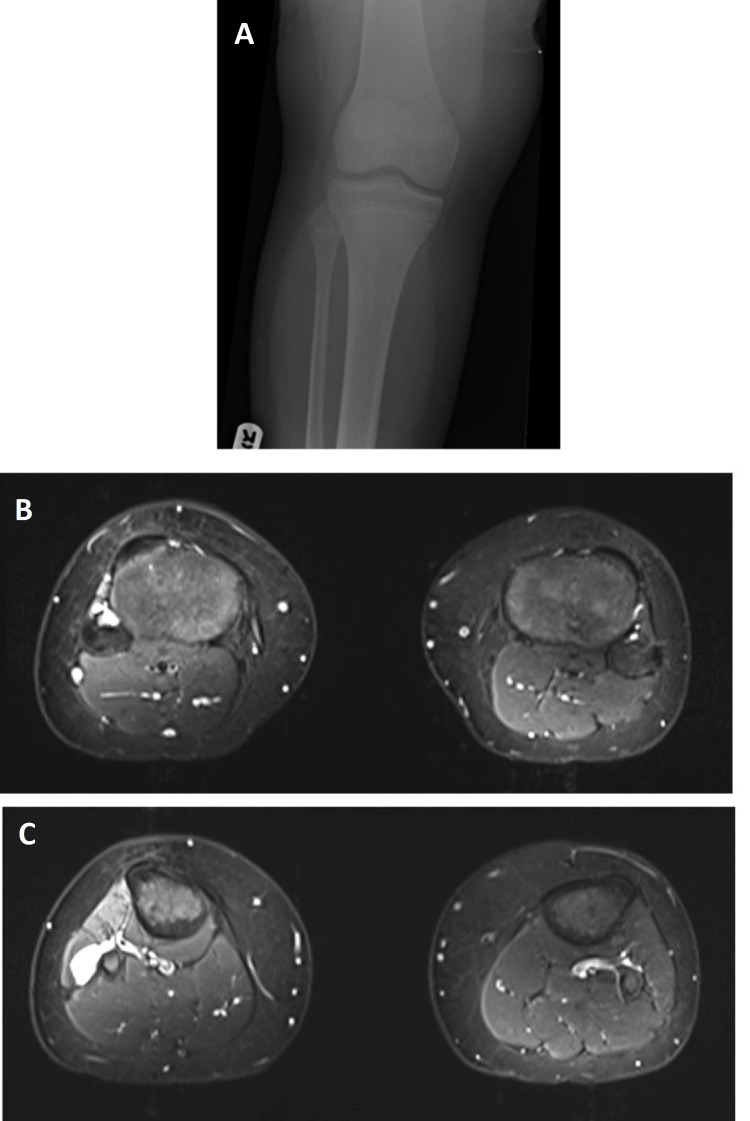
(A) Plain radiographs of the right knee – no bony abnormality seen. (B, C) Axial cuts in T2-weighted MRI images of both legs at the level of proximal tibia showing right cystic lesion in close relation to the CPN and the proximal tibiofibular joint. CPN - common peroneal nerve

Plain radiographs of the knee and lower leg showed no bony lesions. Magnetic resonance imaging (MRI) of the lumbosacral spine was normal. MRI of the knee showed that the CPN was diffusely swollen over a 10 cm distance starting proximal to the lateral head of the Gastrocnemius insertion and extending down to the neck of the fibula where the CPN enters the anterior compartment. Gross edema was demonstrated in the anterior compartment muscles with fatty atrophy seen on the T1 weighted scans in keeping with neuropathic changes. Also, there was a cystic lesion around the Fibular neck suggestive of a possible ganglion cyst that lay in proximity to the inferior aspect of the proximal tibiofibular joint which caused scalloping of the anteromedial aspect of the proximal fibula (Figures 1A-1C).

Nerve conduction studies and electromyogram (NCS/EMG) have suggested the presence of compression affecting the CPN at the level of the Fibular head with active denervation changes and little regeneration. Given those findings, the patient and his parents were counseled for surgical exploration, decompression of the CPN, and excision of the ganglion cyst which was performed on an expedited basis.

Treatment

**Figure 2:**
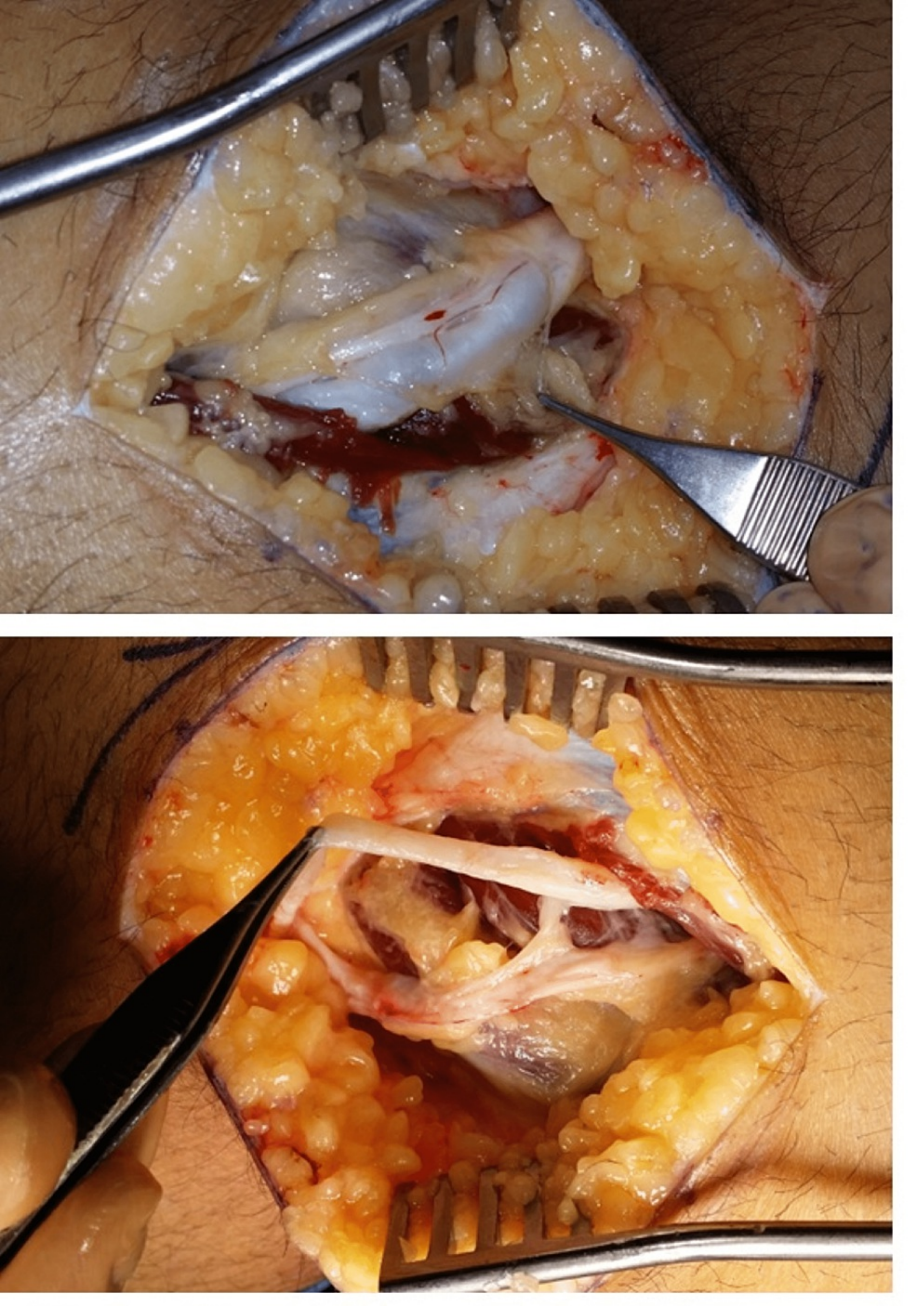
(Top) Lateral approach over the knee showing over the CPN with pressure due to underlying large synovial cyst. (Bottom) CPN dissected and freed from the large ganglion cyst.

Intraoperatively, a large cyst was found under the deep fascia, arising from the proximal tibiofibular joint wrapped around the CPN causing significant compression. The CPN was swollen proximally and stretched over the cyst but in continuity, which was dissected carefully isolating it from the cyst. CPN was freed and dissected both proximally and distally (neurolysis) (Figure 2).

After the nerve decompression, the cyst was traced to the proximal tibiofibular joint, where its sack was tied and excised. The anterior compartment muscles showed positive activity with CPN stimulation using a nerve stimulator intra-operatively. Histological examination of the lesion showed a fibrous thin wall cyst consistent with a large ganglion cyst with several entrapped medium-sized separated nerve fascicles with areas suggestive of neuroma formation.

Outcome and follow-up

Follow-ups at six weeks, three, and six months showed no recovery of the CPN with no dorsiflexion power but with a positive Tinel test over the distal end of the scar. The patient was struggling to use the foot drop splint and therefore discarded it leading to the development of calf tightness and ankle stiffness in an equinus position as a result. Serial casting was performed over 6 weeks which helped to improve his ankle equinus contracture. A repeat NCS/EMG showed subacute axonal degeneration with no regeneration potential. Tibialis posterior muscle activity was good but none in the tibialis anterior muscle.

At one-year post-surgery, there was no active dorsiflexion but a passive range of movement up to 15 degrees of dorsiflexion. As there was no prospect of CPN recovery at that stage, which was estimated to be around three years from his initial symptoms, a tendon transfer procedure was suggested to address his foot drop. This was discussed and approved after a departmental multidisciplinary team (MDT) discussion and surgery was performed around 18 months post-exploration surgery. Tibialis posterior tendon was sutured over the lateral and middle cuneiforms using a bio-tenodesis screw and an anchor. At six weeks postoperatively, the patient had healed wounds and intact functioning tibialis tendon transfer but was still using a temporary foot splint. Physiotherapy was initiated at that stage allowing partial weight bearing. More extensive physiotherapy was required at six months as the patient presented with a recurrence of tightness in his calf muscles and an inability to dorsiflex beyond neutral passively despite having grade 4 power in the transferred tibialis posterior muscle.

At 12 months follow-up, the patient was able to walk reasonably well while wearing appropriate footwear; however, he continued to markedly struggle to walk barefoot due to a residual degree of foot drop. This was attributed to slight weakness in the transferred tendon function, in addition to an element of calf tightness and ankle stiffness. Further discussions were held with the patient and his family regarding a possible third surgery for calf lengthening and advancement of tibialis posterior tendon, however, the patient declined and opted to continue with non-operative management to date. Currently, six years after the index nerve decompression surgery, the patient has not regained his pre-injury nerve function with a remaining degree of persistent foot drop and intermittent neuropathic pain.

## Discussion

CPN becomes vulnerable to injury or compression as it winds around the fibular neck due to its superficial course [2-4,7]. Since Sultan et al. described the first neuropathy in the lower limb in 1921 [2], only a few cases reported in the literature regarding compression neuropathy, particularly caused by a ganglion cyst [3,5-10], most of them are described in adults. Although the prevalence of these types of lesions is quite low [4-6], it is of utmost importance to be included as a differential diagnosis for atraumatic progressive foot drop in both children and adults. CPN palsy can be due to either intraneural or extraneural causes. Multiple etiologies of CPN palsy have been reported in the literature [3-17] as outlined in Table 2. Other causes of foot drop are lumber disc herniation and traumatic CPN injury.

**Table 2:** List of different etiologies reported in the literature CPN - common peroneal nerve, SPN - superficial peroneal nerve

Study	Cause or compression
Edwards et al., 1995 [13]	Extraneural synovial cyst – Lateral meniscus
Rawal et al., 2004 [8]	Extraneural ganglion cyst – proximal tibiofibular joint
Frank et al., 2008 [5]	Extraneural ganglion cyst – proximal tibiofibular joint
Spinner et al., 2008 [6]	Extraneural ganglion cyst – proximal tibiofibular joint
Greer-Bayramoglu et al., 2008 [7]	Extraneural ganglion cyst – proximal tibiofibular joint
Bajuri, 2011 [9]	Extraneural ganglion cyst – proximal tibiofibular joint
Ozden et al., 2013 [3]	Extraneural ganglion cyst – proximal tibiofibular joint
Erdil, 2013 [10]	Extraneural ganglion cyst – proximal tibiofibular joint
Kukreja et al., 2015 [14]	Extraneural ganglion cyst – proximal tibiofibular joint
Cebesoy, 2007 [15]	Intraneural - Giant Plexiform Neurofibroma – CPN
Stamatis et al., 2010 [4]	Intraneural ganglion cyst – SPN
Waldschmidt, 2010 [11]	Intraneural ganglion cyst – CPN
Tehli et al., 2011 [16]	Intra neural ganglion cyst – CPN
Muramatsu, 2013 [12]	Intraneural ganglion cyst – CPN
Schmidt, 2017 [17]	Multiple Hereditary Exostoses with Synostosis of the Proximal Tibiofibular Joint

To our knowledge, all cases reported of CPN palsy were in adults with only one pediatric case reported in the published literature, who developed CPN palsy due to a ganglion cyst from the proximal tibiofibular joint. This was a case report of a 12-year-old child reported by Frank et al. [5]. We described a 16-year-old patient in this report, which is the second case of this nature in the literature.

As regards the intraneural ganglion cysts, Tehli et al. reported an intraneural cyst [16] which was described to be located within the nerve substance causing compression and resulting in CPN palsy. Muramatsu also described an unusual intraneural ganglion cyst, which involved the CPN and its branches causing nerve palsy [12]. Similarly, the extra-neural cysts causing CPN palsy are also extremely rare and only a few cases reported in the literature. Edward et al. reported an unusual case of a cyst arising from the lateral meniscus which extended posterolaterally compressing the CPN [13]. Kukreja et al. [14] more recently reported a case of a patient with CPN palsy due to a ganglion cyst arising from the proximal tibiofibular joint. Similarly, Rawal et al. [8] reported another similar case and they thought the origin of those cysts was due to the articular branch of CPN to the proximal tibiofibular joint. Erdil et al. [10] reported a case of atraumatic sudden foot drop that was due to a synovial cyst. Ganglion cysts can also develop as a result of direct or indirect trauma to the lower limb causing injury to the superior tibiofibular joint [6].

The clinical diagnosis can often be challenging if the ganglion is not easily palpable. Ganglion-associated symptoms are variable but mostly associated with pain radiating along the course of the compressed nerve, altered sensation, paresthesia, and foot drop in delayed presentations. Timely diagnosis and prompt management of a suspicious case after confirmation tests are important; several reports emphasize the importance of early diagnosis which has the utmost implication for complete recovery of the paresis [5].

Plain radiographs have a limited role in establishing the diagnosis of ganglion cysts but may help narrow down the differential diagnosis by excluding any abnormal bony lesions. Electrophysiological studies help to identify the level and severity of the compression and whether it is radiculopathy (root lesion) or a peripheral nerve lesion. When root lesions are suspected, a needle EMG examination of the lumbar paraspinal, gluteal, and hamstring muscles would be very valuable [6,7].

MRI remains the gold standard investigation of choice as it is a noninvasive investigation and allows localizing, sizing the lesion accurately, and analyzing the state of the muscles supplied by the Peroneal nerve. However, It may be difficult to differentiate a ganglion cyst from nerve sheath tumors or solid masses on MRI. Ultrasonography (US) may be effective in showing the cystic nature of the mass (well-circumscribed) and in differentiating it from solid tumors (anechoic lesion) [3,9].

In this case report, there was no history of trauma or extrinsic factors causing nerve compression. Plain radiographs were normal, MRI of the lumbar spine and NCS excluded proximal pathology at the L5 nerve root and localized the pathology around the proximal tibiofibular joint.

Currently, the recommended treatment for CPN palsy due to ganglion compression is the surgical removal of the ganglion with careful pre-operative planning and accurate delineation of the lesion. Local cyst recurrence postoperatively is reported and Rawal et al. hypothesized that the origin of peroneal nerve ganglia is the proximal tibiofibular joint, via the articular branch [8]. Various studies stress the importance of articular branch ligation to avoid this complication [3,7] as simple excision of the ganglia is not sufficient to prevent local recurrence. Other surgical complications reported included nerve traction injuries, perineural fibrosis, and less commonly, complete nerve transection [7].

As with most ganglions, simple aspiration of the cyst yields a high risk of recurrence rates. In addition, the extensive branching pattern of the Peroneal nerve around the neck of the fibula renders the nerve susceptible to damage which probably predicts less favorable outcomes [1,3,4,10,11,12,17].

Fabre et al. reported the results of 60 patients with idiopathic peroneal nerve palsies, who were treated with surgical decompression. Motor function recovery postoperatively was good to excellent in 87% of his series. They recommended surgical decompression even for patients presenting with only sensory symptoms confirmed by electrophysiological studies preoperatively [18].

The prognosis due to compressive pathologies after surgical decompression is quite favorable only if done early but can take a few weeks to recover [10,13]. Malla et al. [19] described in their series of 15 patients who were treated with decompression and neurolysis with a mean follow-up of 42 months that the outcome is usually more favorable if surgery is done within four months after the first presentation of neurological deficits with a mean recovery time of 2.5 months post-operatively. The results were less favorable in patients who have had neurological symptoms for longer than one year [18]. With our patient, it must be noted critically that the presentation was delayed over two years, and so a functional recovery could not be achieved, despite surgical decompression and neurolysis of the CPN. This delay may have led to severe and irreparable axonotmesis of the peroneal nerve rather than demyelination injury which is more favorable.

For cases with irreparable CPN palsy, a tendon transfer procedure is reported in the literature and was first described in 1933 by Ober utilizing the tibialis posterior tendon [20]. This could be done with or without a combined transfer of the flexor digitorum longus and is considered the method of choice for providing a functional recovery of the patients.

## Conclusions

We emphasize the urgency of surgical decompression with neurolysis in the case of CPN palsy. Surgical decompression should be performed as soon as possible and ideally within four months from the onset of the compression. In cases with delayed presentation and established foot drop, caution should be taken when discussing the likelihood of recovery after surgical decompression. Tendon transfer procedures are usually needed and should be offered to patients, who fail to achieve functional recovery or even as a primary salvage procedure in advanced cases with delayed presentation.
